# Some Points in the Treatment of Diabetes—I

**Published:** 1891-09-05

**Authors:** 


### The Practitioner's Mirror and Retrospect.

[*The Editor will be glad to receive offers of co-operation and contributions from members of the profession. All letters should be addressed to* The Editor, The Lodge, Porchester Square, London, W.]

### MEDICINE.

### SOME POINTS IN THE TREATMENT OF DIABETES.—I.

It appears probable that several distinct diseases are included under the common term diabetes, for not only is glycosuria caused by a large number of poisons, and under a great variety of pathological states of different organs, but the process of the formation of the excreted sugar seems to vary essentially, and the course of the disease shows as little similarity in different groups of cases as the course of the different diseases characterised by anæmia or dropsy. If we take the common forms of diabetes, and put on one side for the present the rarer or exceptional cases, as well as all cases of diabetes insipidus, we find they can be provisionally divided under two types. There is the acute incurable disease, seen chiefly in early adult life; and the chronic, milder form, more frequent in the latter half of life, and often co-existing with gouty conditions. Neither type is, however, confined absolutely to patients of a certain age, and there are many cases which cannot be placed under either class, but pursue a course of their own. Thus we may doubt whether even instances of diabetic coma are found in well-defined cases of either type, or whether they form a group of their own. Nor is this merely a question of names and pathological precision, but of grave questions of treatment. In the acute form we are able to do little beyond treating the symptoms as they arise, and putting off the inevitable termination as long as possible. In chronic gouty glycosuria the disease is often as curable as an attack of eczema, but like it, tends to reappear under favourable circumstances. In both forms much improvement is usually obtained by cutting off sugar and starch from the diet. Sugar especially acts not only as a source of glycosuria, but as an acute poison. It is not merely carried over into the urine, but causes the excretion of a far larger amount of saccharine matter than has been actually consumed. Yet even in this question of diet there is considerable difficulty. If a gouty patient develops glycosuria, are we justified in choosing a diabetic diet, and excluding all care for the lithiasis? Is it reasonable to feed such a man on meat, green vegetables, gluten bread, and fats? The sugar may indeed disappear with its attendant discomforts, but what is the effect on the excretion of uric acid produced by a food largely composed of flesh? Some authorities, indeed, contend that such a diet, if the the quantity is carefully regulated, is really beneficial to the gouty state, but it is certainly not that which is usually adopted in gout when uncomplicated with diabetes. If we are undeterred by these considerations, the danger may indeed be minimised by the administration of alkalies and salicylates to remove the uric acid in a soluble form, but the question appears worthy of more consideration than it has hitherto received when it is remembered that these patients may be subjected to the accumulated effects of such a nitrogenous diet continued, perhaps, for twenty or thirty years. Saundby, indeed, confesses that "many elderly diabetics perceptibly decline in health if we enforce those strict rules of diet which nevertheless control their glycosuria most effectually." It does not seem possible then to formulate any hard and fast rule for these cases. Their digestion and gouty symptoms must be watched and the diabetic diet relaxed when necessary.

The most difficult matter in all diabetic diets is the substitute for starch foods. Gluten or almond bread, bran cakes, and similar articles are unsatisfactory. Dujardin Beaumetz is probably right in allowing a certain amount of potatoes in all but the worst forms of the disease, and in two severe cases recorded by Saundby the quantity of urine and of sugar was actually better when potatoes were given in moderate quantities. Fat, and especially cod-liver oil, is usually admissible, and in mild cases milk, though the skim milk diet praised by Donkin, has led to disastrous results, except in a few instances of gouty glycosuria. An important reason for the use of much green vegetables is the frequency of constipation which appears to produce at times very dangerous consequences. Hence also the value of bran cakes. Both substances are also important as giving bulk to the food, and so allaying the excessive appetite which is at times so troublesome. A question of some difficulty has been raised as to the drink, whether or no it is necessary to limit strictly the amount taken. To do so certainly causes much distress, and adds to the difficulty of bearing a rigid diet. It is hard to see what effect it can have on the sugar, and the passage of a large quantity of water is of less importance than that of highly saccharine fluid. Moreover, in gouty and similar toxic cases elimination of the morbid material by washing out the tissues is one of the first objects. "I would flood the tissues of a diabetic," said Saundby, "if I knew how to do so." By placing our patient at first on a rigid diet we may often control the quantity of urine, the thirst, loss of weight, and more or less free the urine from sugar. If this result can be maintained, we may then pass on to the second part of our task, that of "restoring the assimilative power over the carbohydrate elements of food." In a certain proportion this is impossible, but there are others in which the absence of sugar remains after a rigid diet has been given up, and others in which the action of drugs may be added to, or even, after a time, substituted for dieting.

